# How Inflammation Pathways Contribute to Cell Death in Neuro-Muscular Disorders

**DOI:** 10.3390/biom11081109

**Published:** 2021-07-28

**Authors:** Sara Salucci, Anna Bartoletti Stella, Michela Battistelli, Sabrina Burattini, Alberto Bavelloni, Lucio Ildebrando Cocco, Pietro Gobbi, Irene Faenza

**Affiliations:** 1 Department of Biomolecular Sciences (DiSB), Urbino University Carlo Bo, 61029 Urbino, Italy; michela.battistelli@uniurb.it (M.B.); sabrina.burattini@uniurb.it (S.B.); pietro.gobbi@uniurb.it (P.G.); 2 Cellular Signalling Laboratory, Department of Biomedical and NeuroMotor Sciences (DIBINEM), University of Bologna, 40126 Bologna, Italy; lucio.cocco@unibo.it (L.I.C.); irene.faenza2@unibo.it (I.F.); 3 Department of Diagnostic Experimental and Specialty Medicine (DIMES), University of Bologna, 40126 Bologna, Italy; anna.bartoletti2@unibo.it; 4 Laboratory of Experimental Oncology, IRCCS Istituto Ortopedico Rizzoli, 40136 Bologna, Italy; alberto.bavelloni@ior.it

**Keywords:** neuro-muscular diseases, motor neuron disorders, innate immune system, neuroinflammation, cell death

## Abstract

Neuro-muscular disorders include a variety of diseases induced by genetic mutations resulting in muscle weakness and waste, swallowing and breathing difficulties. However, muscle alterations and nerve depletions involve specific molecular and cellular mechanisms which lead to the loss of motor-nerve or skeletal-muscle function, often due to an excessive cell death. Morphological and molecular studies demonstrated that a high number of these disorders seem characterized by an upregulated apoptosis which significantly contributes to the pathology. Cell death involvement is the consequence of some cellular processes that occur during diseases, including mitochondrial dysfunction, protein aggregation, free radical generation, excitotoxicity and inflammation. The latter represents an important mediator of disease progression, which, in the central nervous system, is known as neuroinflammation, characterized by reactive microglia and astroglia, as well the infiltration of peripheral monocytes and lymphocytes. Some of the mechanisms underlying inflammation have been linked to reactive oxygen species accumulation, which trigger mitochondrial genomic and respiratory chain instability, autophagy impairment and finally neuron or muscle cell death. This review discusses the main inflammatory pathways contributing to cell death in neuro-muscular disorders by highlighting the main mechanisms, the knowledge of which appears essential in developing therapeutic strategies to prevent the consequent neuron loss and muscle wasting.

## 1. Introduction

Injury or dysfunctions of the motor neuron, the pheripheral nerve or muscles are correlated to a group of disorders known as neuro-muscular diseases [1,2,3,4], which include motoneuron disorders, diseases of the pheripheral nerve or of neuromuscular junction, and muscle disease such as muscular dystrophies [5,6,7] and myopathies (see Table 1 in which two main diseases for each class have been reported). The innate immune system represents the main defense against infection and is involved in tissue repair by eliminating apoptotic cells and cellular debris [8]. It is known that its excessive activation, as well as its non-resolving, induces an inflammatory response [9] which contributes to the disease’s progression. This review is an update of the role of inflammation in inducing cell death in neuro-muscular disorders by highlighting the main inflammatory pathways and discussing their relationship with the innate immune system. In particular, the review focuses on the crucial role of inflammation correlated to cell death by deepening these aspects of motor neuron diseases. In these pathologies, a motor unit number reduction has been documented [10,11], even if the molecular mediators which lead to inflammation and neuron cell death have been poorly characterized. In particular, two prominent motor neuron diseases, amyotrophic lateral sclerosis (ALS) and spinal muscular atrophy (SMA), have been discussed as highlighting the relationship between inflammation and neuron death.

## 2. Motor Neuron Disorders

Among the neurodegenerative diseases characterized by impaired motoneuron function, are ALS and SMA, the patho-mechanisms of which are largely unknown [12,13,14]. ALS is a fatal neurodegenerative disease characterized by progressive muscle paralysis determined by the degeneration of upper motoneurons in the primary motor cortex, lower motoneurons in the brainstem and in the spinal cord [15]. Genetic susceptibility and environmental exposure contributes to the pathogenesis of ALS which can be sporadic or familial, although the distinction can be difficult to assign [16,17]. Neuron death is probably induced by the inability to manage protein aggregates, mitochondrial instability, excess of oxidative stress, defective axonal transport and glutamate toxicity [18,19]. The latter, also known as excitotoxicity, is characterized by glutamatergic overstimulation of motor neurons leading to neurodegeneration by excessive cytosolic calcium release which involves, at least in part, the activation of inositol 1,4,5-trisphosphate receptors located in the ER membrane [20,21]. ALS also involves abnormalities of metabolism and the immune system, including neuroinflammation in the brain and spinal cord [22,23]. Strikingly, abnormalities of the peripheral immune system, with alterations of T lymphocytes, monocytes, complement and cytokines, appear in the peripheral blood of ALS patients. Motoneuron degeneration leads to progressive muscle weakness and wasting, body weight loss, fasciculation, emotional lability and cognitive dysfunction. Around 50 genes have been identified as being involved in the course of ALS [24,25]. The most common cause of ALS is correlated to the repeat expansion of the intronichexanucleotides (G4C2) in the chromosome 9 open reading frame72 (C9orf72) region of the human chromosome 9 [26]. Furthermore, ALS is characterized by other mutations which most involve the SOD copper-zinc 1 (SOD1) gene [27] and, to a lesser extent, mutations in genes that code for TDP-43, fused in sarcoma (FUS) and others [24,25].

In particular, mutant SOD1 is neurotoxic through multiple mechanisms including protein aggregation, oxidative stress, mitochondrial dysfunction and excitotoxicity. Furthermore, it is pathogenic in familial ALS and, in some cases, in sporadic one. On the other hand, TDP43 and FUS are essential proteins involved in several RNA-processing events, including splicing, transcription and translation. Hyper-phosphorylated and ubiquitinated TDP-43 or FUS deposits lead to inclusion body accumulation in the brain and spinal cord of patients and consequent motor neuron degeneration. Although pathological pathways leading to ALS seem to differ between SOD1, TDP-43 and FUS cases, a common hallmark resides in toxic protein aggregation [24,25]. 

Spinal muscular atrophy (SMA) is a motor neuron degeneration, which occurs in the spinal cord causing progressive weaknesses in the limbs and trunk, followed by muscle atrophy [28]. SMA is an autosomal recessive disease characterized by a variable phenotype. The disease is classified in typeI-III based on age at the onset of the disease and clinical course. Mutations in the survival motor neuron gene (SMN1) on chromosome 5q13 cause this disorder and induce the loss of motor neurons in the ventral horn of the spinal cord and the subsequent weakness and atrophy of skeletal muscles [29,30,31]. The homologous SMN2 gene, coding for the SMN protein, it is not able to compensate the SMN1 gene depletion [28] since it mostly generates non-functional SMN protein. The latter, essential for cell survival, is localized in the cytoplasm, the nucleus and nucleoli [32,33], where it regulates several cellular processes including the assembly of various ribonucleoproteins. However, the molecular mechanism by which suboptimal levels of SMN lead to SMA remains largely unknown [34], but its important roles in maintaining the function of motoneurons and muscles are certain. In fact, SMN protein depletion also alters the homeostasis and functioning of other tissues including the skeletal muscle, heart, autonomic and enteric nervous systems, as well as the lymphatic, bone and the reproductive systems [35]. 

In addition to gene depletion, glia cells contribute to motoneuron degeneration in SMA pathology where abnormal microglia increase and astrogliosis and inflammatory cytokines released from activated astrocytes have also been documented [36,37,38]. Despite the genetic causes of SMA being well known, the mechanisms underlying motor neuron death are still poorly understood. The low levels of SMN might lead to a selective activation of intracellular stress signaling pathways that play a central role in initiating neurodegeneration [39,40].

Therefore, in ALS and SMA, inflammation is a common step which contributes to the progression of neurodegenerative diseases, leading to neuron and muscle death (Table 2). Here, the main pathways, which induce inflammation and consequently neuron death in ALS and SMA, have been analyzed based on data from recent literature and by discussing the involvement of the innate immune system and of specific cellular processes.

### 2.1. Innate Immune System

Neuroinflammation includes cellular and molecular processes which encompass the activation of microglia and astrocytes and the infiltration of peripheral immune cells. Thus, abnormal glial and astrocyte activation, as well as peripheral immune cell infiltration in the brain, is neurotoxic and seems to contribute to ALS and SMA pathology [41,42]. In fact, some studies report that an increase in microglia leads to inflammation in the spinal cord of SMA model mice, even if the main mechanism of its activation remains unknown [38].

In nervous tissue microglia represents the main innate immune cell involved in brain development, maturation and homeostasis. Microglia impairment leads to neurodegeneration, resulting in inflammation and thus contributing to neurodegenerative diseases [43,44]. Gene mutations, directly linked to the immune system response, have recently been reported in ALS and SMA patients [45,46,47,48,49,50,51]. These genes seem to be involved in the immune system dysfunctions which occur in spinal muscle atrophies and could play a role in the initial steps of disease pathogenesis leading to either excessive inflammation or immunodeficiency [52,53]. Gene mutations can induce uncorrect proteostasis, dysregulated RNA metabolism, impaired nucleocytoplasmic transport, mitochondrial dysfunction and altered axonal transport. In particular, mutations in SOD1, TARDBP, FUS and C9orf72 cause aggregate formation and consequent inflammatory responses in microglia and neuron death [47,50,52]. In motor neurons, aggregate accumulation can disrupt the transport of mitochondria to areas of metabolic need, resulting in damage to cells and the eliciting of a neuroinflammatory response leading to further neuronal damage [54]. These aggregates have been observed in sporadic ALS patients and usually contained a wild type (WT) transactive response DNA-binding protein of 43 kDa (TDP-43, encoded by TARDBP). Nuclear TDP-43 can be phosphorylated, mislocalized and aggregated in the cytoplasm [55,56] and can act by activating NF-κB, the transcription factor which regulates proinflammatory substrate activation [57,58]. Moreover, dominant mutations in the RNA-binding protein Fused in Sarcoma (FUS) have been identified as co-activators of NF-κB, and its expression in microglia and astrocytes leads to TNF upregulation and consequent motor neuron death [59,60]. Therefore, aberrant, mutant or misfolded proteins aggregate in the cytoplasm or nucleus of microglia, astrocytes and neurons, leading to cellular organellar damage and neuronal death. For instance, mSOD1 accumulation into astrocytes leads to reactive or activated astrocytes which lose their beneficial effect and take on a detrimental role with aberrant glutamate clearance, mitochondrial disfunctions and reactive microglia [61]. 

Gene mutations, in particular on FUS, also interact with SMN1, the main cause of SMA, providing evidence that SMA and ALS are linked at the molecular level (Table 2) [12]. In fact, NFκB upregulation has been reported in SMA model mice [62] and in SMN-depleted BV2 cells too [63]. This is a consequence of SMN gene deletion, which induces the activation of an E3 ubiquitin ligase (TRAF6) downstream of NFκB and c-Jun NH2-terminal kinase (JNK) signaling. An increased phosphorylation of JNK mediates the neurodegeneration observed in the SMA phenotype [40] which involves caspase 3 activation and apoptotic neuron death [39]. JNK activation regulates various downstream targets which include neuronal development, regeneration and death [39,40,64]. In particular, when JNK phosphorylates c-Jun at serine 63 or 73, it induces the formation of the AP-1 complex and the activation of the c-Jun target gene, responsible for neuronal apoptosis both during physiological and pathological conditions. To date, the role of this pathway appears controversial. Pilato and coworkers [65] demonstrated no evidence of stress-activated JNK-c-Jun signaling in SMA mice or human tissues (thoracic spinal cord samples of SMA patients had been collected during autopsies), while also recognizing its fundamental contribution to normal motoneuron development. Moreover, TNFα, the major proinflammatory cytokine, acting through two main receptors, the p55 TNFα receptor (TNFR1) and the p75 TNFα receptor (TNFR2), is implicated in motor neuron death. TNFR2 expressed by astrocytes and neurons is implicated in motor neuron loss in primary astrocyte-spinal neuron co-cultures [66]. Its upregulation contributes to motor neuron degeneration in SMA pathology by inducing uncontrolled neuronal apoptosis due to the partial loss (about 50%) of NAIP gene (neuronal apoptosis inhibitory protein), as demonstrated in SMA type I patients [67]. 

Thus, NFκB appears the main inflammatory factor, common both in SMA and ALS phenotypes, where it mediates the inflammatory response in microglia. Microglia appears massively activated in the regions of neural loss, determining a condition known as “reactive microgliosis”, and interacting with inflammatory T lymphocytes as well as astrocytes in order to provide inflammatory response [68,69]. Microglia activation in the central nervous system (CNS) is heterogeneous and can develop two phenotypes, known as M1, which promotes the neurodegeneration in atrophic diseases [70], and M2, with a neuroprotective effect (Table 3). The M1 microglia phenotype contributes to NF-κB upregulation mediating the activation of pro-inflammatory cytokines, e.g., IL-6, IL-1β and TNFα, and to the pro-inflammatory enzymes which induce an increase in the levels of reactive oxygen species (ROS) and, consequently, neuron death [71,72,73,74]. 

Astrocytes, on the other hand, are the most abundant glial cells in the CNS, where they modulate ionic homeostasis, the action of neurotransmitters (particularly excitatory ones) and play a crucial role in secreting growth factors and nutrients to help neuronal survival [75,76]. Furthermore, they contribute to regulate blood–brain barrier function, synaptic plasticity and neuroprotection [77,78]. As described for microglia, reactive astrocytes also have two phenotypes, A1 and A2. The first phenotype expresses inflammatory cytokines which are harmful and destructive to synapses, whereas the second one produces neurotrophic factors and help synapse repair [79]. An excessive or disproportionate innate immune response promotes neuronal damage and chronic neuroinflammation, in particular that which is detected in the early stages of ALS and SMA [69,80,81]. Motor neuron degeneration is strictly related to the abnormality of the immune peripherical system with dysfunctions in T lymphocytes, monocytes and the complement system. There is some evidence which shows how genetic removal of T lymphocyte cells accelerated disease progression with the upregulation of proinflammatory cytokines in mSOD1 mice, while reconstitution of T lymphocyte cells seems to favor the survival of mSOD1 mice whilst inhibiting the activation of M1 microglia [82]. In SMA mouse models some alterations in T lymphocyte cell maturation or development have been reported and seem to result from an abnormal neuroinflammatory response and the exacerbation of disease [37]. Motor neuron loss is also mediated by monocyte activation which is characterized by a reduced phagocytosis and other dysfunctionalities. These cells have been found both in the blood and in the spinal cord of ALS patients [83]. Finally, even if the role of the complement pathway in ALS pathogenesis is still controversial, the complement system activation may precede end-plate denervation in human ALS [82]. Moreover, the complement system seems to play a central role in SMA disease, where its deregulation leads to the disruption of neuronal networks with consequent synaptic elimination by aberrant activated glia cells and compromised neuronal function. The classical complement pathway mediates the microglia-dependent remodeling of the spinal motor circuits during development and in SMA [84]. 

**Figure 1:**
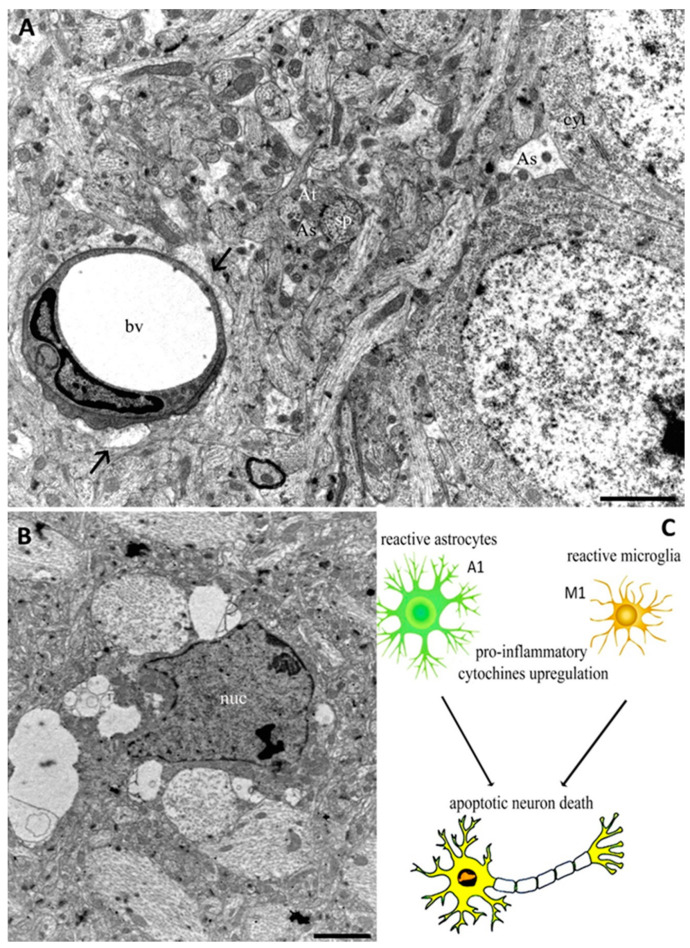
Transmission Electron Microscopy (TEM) images from cerebral cortex of adult rat (**A**,**B**). In (**A**), astrocyte end feet protrusions (arrows) can be observed near to a blood vessel (bv). Astrocyte processes (As) appear near axonal terminal (At) and dendritic spines (sp), as well as around neuron cytoplasm (cyt). The micrograph (**B**) shows a microglia cell characterized by a nucleus (nuc) with an irregular outline in which the chromatin is clumped beneath the nuclear envelope. The schematic representation of glia activation related to neuron death appears in (**C**). Bars 2 µm for (**A**) and (**B**).

Therefore, under stress conditions, such as progressive neurodegeneration, both microglia and astrocytes appear activated, leading to detrimental outcomes in neuronal cell function due to the excess production of different neurotoxic cytokines and noxious molecules [68,85]. Figure 1 shows the main players of the innate immune system in the CNS, such as astrocytes and microglia, highlighting their morphology and localization. In addition, Figure 1C shows glia cell involvement in inducing neuron death. Thus, when glia cells are activated by genetic mutations or pro-inflammatory stimuli, they acquire a reactive phenotype and release pro-inflammatory components responsible for neuron degeneration.

### 2.2. Cellular Mechanisms

Furthermore, cellular mechanisms, such as mitochondrial dysfunction, proteasomal and autophagic impairment, contribute to neuroinflammation and therefore to neuron degeneration [86,87,88,89,90]. In fact, other genes such as p62/SQSTM1, TBK1, OPTN, ALS2, CHMP2B, C9orf72 and CYLD appear activated in these diseases, where they enhance protein aggregation or misfolding with the impairment of proteasomal or autophagosome degradation and consequent inflammation [90,91,92,93,94,95]. In particular, mitochondria play a crucial role in the choice of neuron fate since they are the intracellular organelles mostly involved in the regulation of both cell survival and cell death [96,97,98]. Furthermore, some research revealed that this organelle plays a central role in modulating both innate and adaptive immune responses, thereby providing a link between neurodegenerative and neuroinflammatory processes [89,99,100]. In the CNS, energy production is essential for neuron communication. The large ATP amount is guaranteed from mitochondria which, in order to maintain a correct morphology and function, require the involvement of several processes such as: the synthesis of outer and inner mitochondrial membrane components and mitochondrial proteins, the synthesis and import of proteins encoded by the nuclear genome, lipid import, oxidative phosphorylation, the replication of mtDNA and mitochondrial fusion and fission [98,100,101]. In order to maintain mitochondrial biogenesis, the nuclear genome and the mitochondrial genome must work in a coordinated manner [101] to eliminate the risk of mitochondrial dysfunction and an increase in oxidative stress [102]. Furthermore, fusion and fission events are involved in regulating and controlling mitochondrial dynamics [103]. In fact, during fission process, defective mitochondria are removed by mitophagy [104], while healthy mitochondria selection is based on the number of mtDNA copies, optimum matrix metabolites, and components of the mitochondrial membrane [103,105]. Therefore, alterations in the processes which control mitochondrial biogenesis underlie neurodegeneration, resulting in neurodegenerative diseases, including ALS and SMA [106,107,108]. Mitochondrial complex deficiencies lead to protein hyperphosphorylation, such as the mutant SOD1, and proteosome activity impairment [98]. Alterations in mitochondrial morphology and dynamics observed in the pathogenesis of ALS seem due to high levels of free radicals and oxidative stress, as well as to the modification of the amount of proteins involved in mitochondrial fusion and fission. These abnormal processes induce both caspase-dependent and -independent apoptotic cell death directed by mitochondria, where the mutant SOD1, considered one of most common causes of ALS, plays a central role by interacting with the Bcl-2 protein family [109]. In particular, it has been observed that SOD1 mutations induce mitochondrial respiratory deficiencies [110,111]. In fact, in normal conditions this gene works as an antioxidant enzyme protecting neurons from the free superoxide radical. Therefore, its mutated form stimulates protein aggregation and apoptosis. Furthermore, mutant SOD1 mediates the alteration of the mitochondrial calcium-loading capacity, the impairment of electron transport chain activities, the increase in aberrant ROS production [112,113]. In addition, it blocks protein import at the mitochondrial outer membrane and the antiapoptotic actions of the Bcl-2 protein [98]. 

In SMA pathology, mitochondrial dysfunction, oxidative stress and the impairment of bioenergetic pathways could be correlated to SMN deficiency. High free radical levels have been observed in an SMA cell line and and in several SMA models, where dysfunctional mitochondria, an increase in cytochrome c oxidase activity, the potential loss of the mitochondrial membrane and altered axonal mitochondrial transport have been detected [114,115]. 

**Figure 2:**
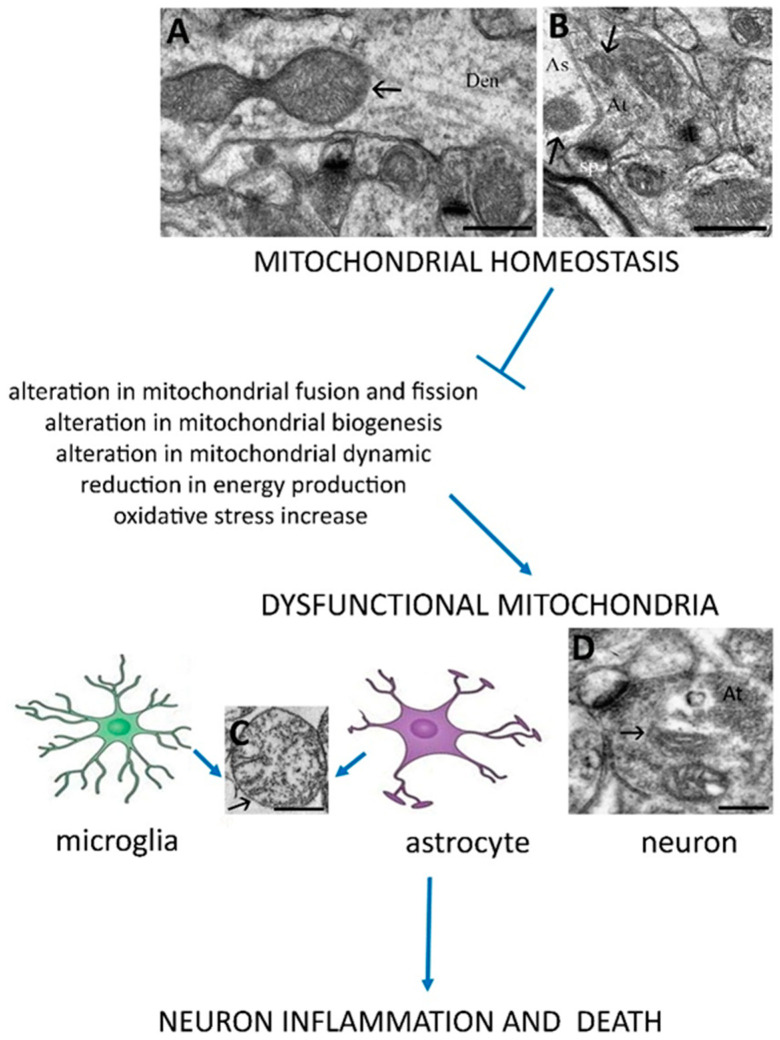
The scheme suggests that preserved mitochondrial processes such as correct biogenesis, fission and fusion, dynamics and other processes are necessary to reach neuron homeostasis. On the other hand, their deregulation in glial cells or neurons contributes to neuroninflammation and death. TEM images, obtained from an adult rat’s cerebral cortex, show mitochondria (arrows) in a dendritic cell (Den) during a fusion event (**A**), in an axon terminal (At) and in an astrocyte process (As) which surrounds a synapse (**B**). Altered mitochondria (black arrows) appear in (**C**,**D**). Bars: 500 nm for (**A**–**C**); 200 nm for (**D**). sp: dendritic spine.

Mitochondrial alterations, which affect microglia, astrocytes and neuron cells, are especially detrimental for neuron survival due to a low neuronal regeneration capacity. Therefore, mitochondrial dysfunctions play a central role in the pathogenesis of neurological disorders. Figure 2 shows mitochondrial morphology in a homoeostatic condition, where the subcellular organelles, localized into astrocytes, dendritic cells and axon terminals, present a preserved cristae rearrangement and, sometimes, fusion mitochondria morphology can be observed, suggesting a cellular function preservation. 

Alterations in mitochondrial morphology and dynamics lead to important implications for cellular function. In particular, dysfunctional mitochondria on resident cells, such as microglia and astrocytes, reduce their role in supporting neuronal survival, synaptic functions and local immunity, thus promoting inflammation and cell death [116].

SMA pathology is primarily characterized by apoptotic motor neuron death in the spinal cord, mediated, at least in part, by mitochondria [117,118]. These organelles activate the intrinsic pathway of apoptosis [119], which involve a combination of pro- and anti-apoptotic proteins, including Bcl-2 family members, caspase family members, cytochrome c and p53. Several studies have shown altered levels of these proteins in patients and models of SMA [120]. Moreover, the NAIP gene, located within the genomic region of chromosome 5 that encompasses SMN1, plays a crucial role in neuron fate. In fact, mutations of NAIP, a gene previously described in the activation of the immune system, induce and increase in the release of caspases 3 and 9 during apoptosis, with deleterious consequences for SMA motor neurons and, thus, an acceleration in the progression of the pathology [118,121,122]. Different apoptotic pathways are responsible for motoneuron loss both in ALS and SMA. In the Drosophila S2 cell model, SMN loss activates the caspase-dependent apoptotic signaling, with upregulation of Fas ligand-mediated apoptosis and of caspase 8 and 3 [123,124]. A critical regulator role in neuronal apoptosis has been associatedwith the Bcl-2 family proteins. In particular, in muscle atrophy diseases the Bcl-2 anti-apototic proteins, such as Bcl-2 and Bcl-X, appear downregulated by enhancing neuronal apoptosis [124,125,126,127,128]. Moreover, the nucleolus accumulation of p-53, a protein which regulates neuronal apoptotic death and usually binds to the SMN protein (when it is not truncated), contributes to the activation of apoptosis in motor neuron. Finally, MAP kinase cascades, implicated in the modulation of proliferation, differentiation, apoptosis or survival, inflammation and innate immunity, also regulate neuron apoptosis. This pathway acts by activating mitogen-activated protein kinases (MAPKs) such as JNK, p38 MAPK and TNF-α pathways, and their upregulation contributes to the pathology of neuro-muscular disorders [63,69]. 

Therefore, maintaining mitochondria homeostasis in the CNS is a prerequisite to avoid the excessive generation of ROS and cell death and could represent the starting point for the treatment of inflammatory motor neuron diseases. The main mechanisms involved in achieving mitochondrial functional homeostasis, are the ubiquitin-proteasome system, which specifically targets unfolded proteins [6,129], and mitophagy, which controls the mitochondria’s quality and can represent the possible therapeutic target for alleviating cell death and nervous tissue injury [130]. When misfolded aggregated proteins, which are one of the features of neurodegenerative disorders, accumulate in the cytoplasm, they activate the unfolded protein response (UPR) pathway. Among the stress sensors involved in UPR activation, an important key regulator is the protein kinase R (PKR). It is a member of a family of serine-threonine kinases that phosphorylate the translation initiation factor in its subunit [131,132]. This inhibits most cellular translation and selectively promotes the translation of cytoprotective genes that enhance the protein folding or degradation of misfolded proteins. When the UPR is overwhelmed by misfolded proteins and is unable to adequately protect the cell during times of stress, cellular apoptosis and autophagy are induced [133]. This particularly occurs in ALS, which is characterized by the abnormal accumulation of misfolded or aggregated proteins, and therefore the dysfunction of proteostasis which significantly increases endoplasmic reticulum stress and leads to neuronal degeneration [134,135].

A key role in mitochondrial homeostasis is played by mitophagy, which can be initiated via PINK1 (PTEN induced putative kinase 1) and PRKN (parkin RBR E3 ubiquitin protein ligase) -dependent and -independent pathways, through which damaged mitochondria are selectively degraded by lysosomes [136]. In particular, altered mitochondria are not able to import PINK1 into the inner mitochondrial membrane, leading to the accumulation of PINK1 on the outer mitochondrial membrane which cause the activation of ubiquitin or PRKN, linking phosphoubiquitin chains to various mitochondrial surface-associated proteins. The latter are recognized by cargo-receptor proteins such as SQSTM1/p62 (sequestosome 1), OPTN (optineurin) and NBR1 (NBR1 autophagy cargo receptor) that subsequently interact with MAP1LC3B/LC3B (microtubule associated protein 1 light chain 3 beta) to start autophagosome formation [137]. Intriguingly, these receptors can be the target of activated TBK1 (TANK binding kinase 1), which enhances mitophagy by phosphorylating autophagic receptors, especially OPTN [138,139,140]. Several authors have been demonstrated that the loss of function of OPTN or TBK1 or mutations in some genes such as SQSTM1 result in impaired mitophagy which leads to the accumulation of damaged mitochondria. This impairment, characterized by the inefficient turnover of mitochondria, has been identified in different ALS models. Thus, mitochondrial dysfunction and accumulation can be considered a prevalent feature in the motor neuron degeneration of ALS patients [137,139,141].

Furthermore, TDP-43 seems involved in the alteration of several mitochondrial pathways such as mitochondrial dynamics, mitochondrial trafficking and energetic metabolism [142]. In fact, ALS patients show high amounts of TDP-43 aggregates in mitochondria, which induce mitochondrial damage and cell death. On the other hand, blocking the link between TDP-43 and mitochondria is sufficient to prevent the neuronal loss and mitochondrial dysfunction [143,144]. Therefore, mutations in several genes known to contribute to ALS result in the deposition of their protein products as aggregates, by altering the correct mitophagy, resulting in damage to cells, and thus eliciting a neuroinflammatory response leading to further neuronal damage and death. As described above, impaired mitochondrial biogenesis has been reported in the muscle of SMA patients as well as in the motor neurons of SMA mice too [87,145]. In particular, in SMN-deficient spinal motoneurons, a dysregulation in mitochondrial bioenergetics-related genes, a reduction of ATP synthesis and oxidative stress increase have been described by several authors [87,146,147]. Furthermore, an impairment of the autophagic degradative pathway has been observed in cell culture and animal models of SMA too. In vitro studies in SMN-depleted cells demonstrated increased p62/SQSTM1 protein levels suggesting a decreased autophagic flux [148,149]. On the other hand, Piras et al. demonstrated that the inhibition of autophagy suppressed autophagosome formation and reduced the apoptotic activation [150]. Therefore, to date, the role of autophagy/mitophagy in SMA disease remains controversial and should be further investigated. 

## 3. Conclusions

**Scheme 1:**
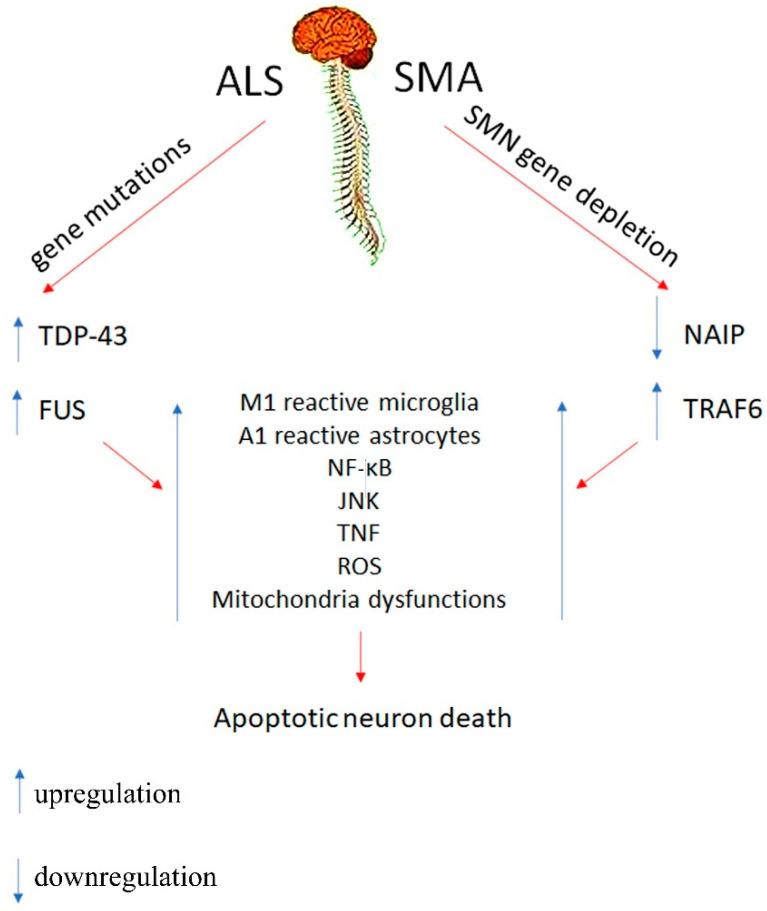
The scheme shows the main cellular factors which contribute to neuroinflammation and lead to apoptotic neuron death in SMA and ALS diseases.

Neuro-muscular disorders are diseases that affect the development and growth of the neuro-muscular system [4,151,152]. The pathology can appear anywhere along the neuro-muscular pathway; in the brain, the nerves and muscle fibers [152]. Although there is a recognized multitude of neuro-muscular disorders, this review describes the role of inflammation in motor neuron disorders by highlighting the involvement of the main pathways which lead to neuron death. ALS and SMA are a gene group of acquired or inherited rare disorders caused by injury or dysfunction of the anterior horn cells of the spinal cord (lower motor neurons) with severe consequences to the peripheral nerves, neuromuscular junctions, or skeletal muscles leading to muscle weakness and wastage [36,153]. In mouse models of several neuromuscular diseases, apoptosis signaling, which greatly contributes to pathology, appears activated by brain injury, including neuro-inflammation, damaged mitochondria and altered degradative processes. These alterations induce neuron cytotoxicity and death, resulting in neurological dysfunctions. For instance, ALS pathogenesis seems to be caused by numerous interactions of molecular and genetic pathways. Several mutations are linked to ALS. To date, the main mutations have been detected in the SOD1, C9ORF72, TARDBP and FUS genes which lead to dysregulated RNA metabolism, with the formation of intracellular neuronal aggregates that have been detected in glia or neuron cell of ALS models. Mutations in the SOD1 gene increase oxidative stress by inducing mitochondrial dysfunction and defective axonal transport. Furthermore, impaired glutamate uptake from the synaptic cleft induces glutamate excitotoxicity due to the dysfunction of the glial excitatory amino acid transporters. All these factors trigger neurodegeneration and, among the mechanisms implicated in motor neuron disorder development, innate immune system alterations seem to play a crucial role in mediating neuronal damage [154,155]. It is known that glia cells play a crucial role for the immunity, neurogenesis, synaptogenesis, neurotrophic support and phagocytosis of cellular debris, as well as helping to maintain CNS integrity and homeostasis [155,156]. Activated microglia and astrocytes have the capacity to release proinflammatory mediators leading to neuroinflammation that, when they become uncontrolled, can give rise to various neurological disorders including ALS and SMA [155]. M1 microglia and A1 astrocyte uncontrolled activation leads to the inflammation and upregulation of inflammatory pathways. Both in ALS and SMA, the common inflammatory pathway requires the activation of NF-κB, which induces the upregulation of downstream cytochines leading to apoptotic cascades and neuron death. Moreover, a crucial role in disease progression and development is related to mitochondrial alterations and autophagic impairment [87,99,157,158,159], which create aggregates of abnormal proteins with potential toxicities and cause inflammation response generation and finally apoptotic cell death (Scheme 1). As a consequence, due to the inability to get rid of the irregular aggregate proteins, neurons are unable to properly function and begin to degenerate. Therefore, the process of modulating neuro-inflammation and maintaining a correct mitochondrial function and morphology could represent an effective therapeutic target for ALS and SMA treatment. 

## Figures and Tables

**Table 1:** Classification of neuro-muscular diseases.

Motor Neuron Disorders	Pheripheral Nerve Diseases	Neuro-Muscular Junction Diseases	Muscular Dytrophies	Myopathies
Amiotrophic Lateral Sclerosis (ALS)	Charcot-Marie-Tooth disease (CMT)	Myastenia Gravis (MG)	Duchenne Muscular Dystrophy (DMD)	Inflammatory Myopathies
Spinal muscle atrophy (SMA)	Giant Axonal Neuropathy	Congenital Myasthenic Syndromes (CMS)	Becker Muscular Dystrophy (BMD)	Metabolic Myopathies

**Table 2:** Similarities between ALS and SMA.

ALS and SMA Similarities
Neuroinflammation
Motor neuron death
Common molecular pathways
Loss of muscle mass and strenght
Weakness, breathing difficulties
Muscle atrophy

**Table 3:** Factors regulating M1/M2 microglia switching. Pro-inflammatory (Il-17, IFNγ, TNF-α) and anti-inflammatory (IL-4, IL-10, TGF-β) stimuli.

M1 Neuroinflammation Induction	M2 Homeostasis Mainteinance
IL-17	IL-4
IFNγ	IL-10
TNF-α	TGF-β
Inflammatory cytochine production	anti-inflammatory molecule production
